# Biologics in asthma management – Are we out of breath yet? 

**DOI:** 10.5414/ALX02192E

**Published:** 2021-02-12

**Authors:** Nadja Struß, Jens M. Hohlfeld

**Affiliations:** 1 Fraunhofer Institute for Toxicology and Experimental Medicine ITEM,; 2 Department of Respiratory Medicine, Hannover Medical School,; 3 Biomedical Research in Endstage and Obstructive Lung Disease Hannover (BREATH), German Center for Lung Research, Hannover, Germany

**Keywords:** severe asthma, biologics, monoclonal antibodies, eosinophilic asthma, neutrophilic asthma

## Abstract

The biologics authorized for the add-on therapy of severe asthma are monoclonal antibodies (mAbs). Before they are considered for therapy intensification, the patient’s asthma endotype is determined on the basis of phenotypic characteristics. So far, 5 biologics are available that target the signaling pathways of the “T_H_2-high” asthma endotype, in which cytokines of the inflammation cascade mediated by type 2 T-helper cells are upregulated. The corresponding phenotype of this inflammatory endotype is severe eosinophilic asthma, with elevated eosinophils, immunoglobulin E, and fractional exhaled nitric oxide (FeNO). In contrast, the heterogeneous “T_H_2-low” endotype is not yet sufficiently understood. Frequently described in this variant is an increase of sputum neutrophils and an increased expression of the T_H_17-mediated interleukin-17 signaling pathway. There are numerous biologics currently in clinical trials, the thymic stromal lymphopoietin (TSLP) mAbs in particular have shown promising results independent of the asthma phenotype.

## Introduction 

For severe uncontrolled asthma, various biologics are available as an add-on therapy to high-dose treatment with inhaled glucocorticoids and additional controllers and relievers (leukotriene receptor antagonists, long-acting inhaled β_2_-agonists). In adults, severe asthma is present when treatment according to GINA levels 4 or 5 is required and symptom control is not achieved despite treatment adherence [1, 2]. Based on this definition, epidemiological studies from the Netherlands and Sweden report a prevalence of severe asthma of 3.6% [3] and 4.2% [4], respectively, of all asthmatics studied. 

Risk factors for severe asthma in adulthood include female gender, persistent eosinophilic inflammation, nasal polyps, sinusitis, analgesic asthma, and recurrent respiratory infections [5, 6, 7, 8, 9]. Persistent uncontrolled asthma promotes increased exacerbations and persistent, sometimes irreversible bronchial obstruction. However, much more common than severe refractory asthma is inadequate symptom control due to external circumstances such as incorrect inhalation technique, lack of treatment adherence, persistent exposure to allergens or pollutants, or even an inaccurate diagnosis [2]. These causes should be considered before intensifying therapy with biologics. 

The first asthma biologic anti-IgE (omalizumab) was approved 15 years ago, paving the way for individualized management of severe asthma in 2005. Five monoclonal antibodies are now available for the treatment of severe asthma. Given the increasing diversity of therapeutic interventional options, the success of biologic treatment depends on the careful selection of patients who have a good chance of benefiting from the chosen drug based on their specific inflammatory endotype. Meta-analyses of randomized controlled trials demonstrate an average 50% reduction in exacerbation rates with all biologics approved for asthma when the appropriate patient population is carefully selected [10]. Casuistically, impressive improvements can be achieved. 

## Endotype determination 

**Figure 1:**
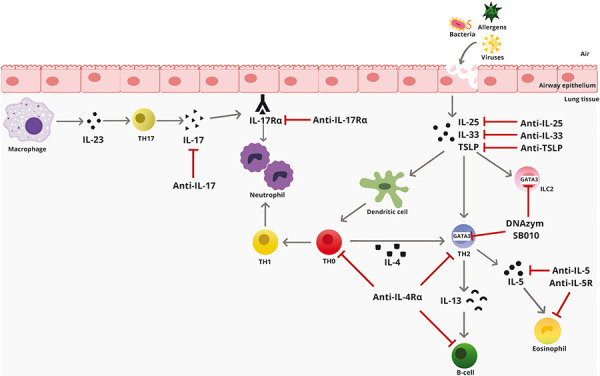
T_H_2-high and T_H_2-low signaling pathways and their inhibitors. IL = interleukin; TSLP = thymic stromal lymphopoietin; ILC = innate lymphoid cell; TH = T helper cell; R = receptor. Arrows: Release of cytokines or activation of cells; T-lines: Inhibition of target structure. Figure in the public domain created with canva.com. Macrophage: by giemsadye licensed under CC BY-SA4.0. Bacterium and virus: by SVG Repo licensed under CC0.

Before starting add-on therapy with biologics, the endotype of the severely asthmatic patient is determined, since the currently approved monoclonal antibodies only address targets of those inflammatory reactions mediated by type 2 T-helper cells (T_H_2 cells) (Figure 1). T_H_2 cells support defense against extracellular pathogens and promote clonal expansion of allergen-specific B lymphocytes. After antigen presentation by dendritic cells, polarized T_H_2 cells secrete pro-inflammatory cytokines, including interleukin (IL)-4, -5, and -13. Under the influence of IL-4, immunoglobulin class switching from IgM to IgE occurs in B cells. In the so-called “T_H_2-high” endotype, T_H_2 cells are increasingly detectable in the airways [11] and drive the eosinophilic inflammatory response. Using phenotypic characteristics and biomarkers, the T_H_2-high endotype can be identified [2]:

- Eosinophils ≥ 150 µL in blood and/or
- FeNO ≥ 20 ppb and/or
- Eosinophils ≥ 2% in sputum and/or
- Allergic asthma with elevated total/ specific IgE.
- Long-term therapy with oral corticosteroids (OCS).

In patients with uncontrolled severe eosinophilic asthma, therapy should initially be intensified by increasing the dose of inhaled corticosteroids (ICS) for 3 – 6 months. If symptom control is inadequate, therapy with an appropriate monoclonal antibody may be considered. Selection must take into account sensitization, blood eosinophils, IgE levels, exacerbations, FeNO levels, comorbidities, and pharmaceutical form. [Table Table1]

## Approved biologics 

### IgE antibody 

Omalizumab was the first monoclonal antibody approved for the treatment of severe allergic asthma in the EU in 2005. It binds to IgE and thus prevents its binding to the FcεRI receptor, which is expressed in particular on basophils and mast cells. The total amount of IgE released is reduced by omalizumab. To determine the correct dose for a patient, the patient’s total serum IgE is determined prior to initiation of therapy. Omalizumab is suitable for asthmatics with, in particular, perennial allergen sensitization, exacerbations in the previous 12 months, total IgE levels up to 1,500 IU/mL, blood eosinophils ≥ 260/mL, and childhood-onset asthma. The agent reduces the number of exacerbations and hospitalizations [12]. 

### IL-5 (receptor) antibodies 

Mepolizumab and reslizumab bind to IL-5, which plays a critical role in eosinophil activation. Similarly, the drug benralizumab blocks the IL-5 receptor on eosinophils and basophils. These agents are therefore suitable for asthmatics with marked eosinophilia of at least 300/mL, rather low IgE levels, late-onset asthma, and frequent exacerbations requiring treatment with systemic glucocorticoids. They reduce the exacerbation rate by 50-75% as well as the need for oral corticosteroids [13, 14, 15]. Mepolizumab is also effective in increasing the remission rate in Churg-Strauss syndrome, which manifests pulmonary as severe allergic asthma [16]. A significant reduction in exacerbation rate with reslizumab is achieved only in patients with a blood eosinophilia of > 400/mL [14]. All antibodies directed against IL-5 reduce the number of eosinophils in blood and sputum. Although eosinophil granulocytes are utilized in the defense against parasites, especially worms, an increase in such infections has not been observed with treatment, although the relevant studies were conducted in countries with a low prevalence of helminth infections [17]. 

### IL-4/IL-13 receptor antibodies 

A biologic that inhibits both IL-4 and IL-13 signaling by binding to the α-subunit of the IL-4 receptor is dupilumab. It is indicated for blood eosinophils ≥ 150/mL, FeNO > 25 ppb and proves effective in both early- and late-onset asthma. Isolated blockade of IL-4 or IL-13 has been shown to be ineffective. Presumably, this might be because on the one hand IL-4 expression is low in asthmatics anyway, and on the other hand inhibition of IL-13 alone addresses bronchial wall remodeling and hypersecretion rather than eosinophil-driven exacerbations, which are often chosen as endpoints in clinical trials [17]. Additionally, dupilumab is approved for the treatment of atopic dermatitis. 

All of the listed biologics reduce exacerbation rates in the appropriate patient population; mepolizumab, benralizumab, and dupilumab also reduce cortisone use with high evidence [10], as does omalizumab, but only modestly [12]. Also, although a slight improvement in quality of life was shown for all five compounds in meta-analysis of randomized controlled trials, the effect on asthma symptomatology and forced expiratory volume in 1 second (FEV_1_) is moderate [10]. However, in individual cases, lung function may increase dramatically and β_2_-mimetic use may decrease, leaving patients significantly fitter in daily life. The attempted treatment with a biologic should be carried out for at least 4 months; if the response is good, the treatment can be continued under continuous re-evaluation, although long-term studies on safety and efficacy beyond 12 months are still lacking. 

## Biologics in clinical trials 

The biologics approved for asthma treatment target cytokines or immunoglobulins that are secreted during T_H_2 cell activation. Inhibition of these mediators, which are at the end of the inflammatory cascade, has the hypothetical and practical advantage of reducing adverse drug effects. The disadvantage, however, is that they can only be used very specifically in selected subgroups of severe asthmatics, as described above. If, on the other hand, blocking occurs at the beginning of the inflammatory cascade, more downstream pathways are affected in parallel, as shown in Figure 1. This presumably increases the effectiveness of the intervention and could address more asthma endotypes. 

For example, inhibitors of the cytokine thymic stromal lymphopoietin (TSLP) follow this principle. Like IL-33 or IL-25, TSLP is an alarmin that airway epithelial cells secrete in response to contact with pathogens or noxious agents. It activates dendritic cells and induces differentiation of naïve T cells to T_H_2 cells, is more detectable in asthmatics, and correlates with disease activity [18]. Tezepelumab is a human IgG2λ antibody directed against TSLP, which was administered intravenously and subcutaneously in phase II studies and reduced bronchial hyperresponsiveness, FeNO, eosinophils, and the number of exacerbations [19]. Tezepelumab inhibited both early and late allergic reactions in mild allergic asthma after inhaled allergen provocation, expressing the potential range of effects [20]. Because the reduction in exacerbation rate was demonstrated in severe asthmatics regardless of phenotype, the Food and Drug Administration (FDA) classified tezepelumab as a “breakthrough therapy” [21]; this status allows for fast track drug development and regulatory review in the United States. Currently, the compound is in phase III as a subcutaneous injection (NCT03927157). 

Another TSLP antibody in clinical development is CSJ117, which has completed phase I. CSJ117 was inhaled once daily for 12 weeks by patients with mild allergic asthma who demonstrated an early and late response after inhaled allergen challenge (NCT04410523). Inhaled anti-TSLP was shown to significantly inhibit the late and early allergic response compared to placebo [22]. 

In addition to TSLP, other alarmins, such as IL-25 and IL-33, are also released by bronchial epithelial cells upon contact with pathogens. IL-33 belongs to the IL-1 cytokine family and plays an important role in innate type 2 immunity by activating eosinophils, basophils, mast cells, macrophages, and innate lymphoid cells (ILC2) through binding to its membrane-bound receptor ST2 (interleukin 1 receptor-like 1). A phase 2 proof-of-concept study demonstrated an improvement in asthma control over placebo (NCT03387852). IL-25 is a member of the IL-17 family and, like TSLP, is involved in the regulation of T_H_2 and ILC2 cells. In asthmatics, the expression of IL-25 is increased [23], and the IL-25 monoclonal antibody ABM125 inhibited the bronchial eosinophilic inflammatory response and reduced the expression of T_H_2-associated cytokines in preclinical studies [24]. Their expression is regulated by GATA3, a transcription factor that plays a critical role in the initiation of T_H_2-driven inflammation and is inhibited by corticosteroids. The inhalable DNA enzyme SB010 inactivated GATA3 mRNA and reduced early and late responses after inhaled allergen provocation in a phase II study in patients with allergic asthma and sputum eosinophilia [25]. Further clinical development with an optimized nebulizer system is planned. [Table Table2]

While omalizumab, the first monoclonal antibody, was approved for severe asthma in 2005, there is no approved personalized pharmacotherapy for the treatment of severe asthma of the “T_H_2-low” endotype to date. The pathomechanisms underlying this heterogeneous endotype are not yet adequately understood. By quantifying granulocytes in induced sputum, neutrophilic, mixed-granulocytic, and paucigranulocytic asthma have been described in addition to eosinophilic asthma [26]. Possibly, IL-17 secreted by T_H_17 cells is a key mediator in neutrophilic asthma, as it has been found to be increased in patients with severe symptoms [27]. IL-17 ensures the integrity of the airway epithelium by regulating neutrophil recruitment after contact with pathogens. However, this property could equally suggest that IL-17 has a protective function and is not a driver but the consequence of asthma [28]. The lack of symptom improvement in subjects with uncontrolled asthma treated with the IL-17 receptor inhibitor brodalumab could point in this direction of interpretation [29]. However, the study population was not screened for neutrophils in sputum or mediators of the IL-17 pathway. Also, in a follow-up study (NCT01902290), which was stopped early due to lack of efficacy, the study population was not selected based on matching endotype. Experience with IL-5 antibodies in clinical trials has shown the importance of patient selection to demonstrate any effect at all [28]. 

A direct IL-17 inhibitor in clinical development is CJM112, which has already been studied in a phase II trial in patients with uncontrolled severe asthma and low blood IgE and eosinophil levels (NCT03299686). The study has been completed, results have not been published yet. 

## Outlook 

The growing choice of biologics as an add-on therapy for severe uncontrolled asthma requires the implementation of endotype determination in clinical practice. For patient selection and monitoring of treatment success, established and readily available biomarkers such as eosinophils and FeNO should be used. However, these are not very meaningful for the “T_H_2-low” endotype; thus further predictors of response to future biologics need to be found. 

## Summary 

“In the future, perhaps we will be able to say ‘[the patient has] an asthma syndrome characterized by mutation sin the IL-X pathway leading to excessive neutrophil chemotaxis in response to pollutants, with secondary structural airway changes’.” Pavord et al. [30] wrote in 2018. In response to the marked endotypic heterogeneity of asthma, biologic therapy as a component of individualized medicine requires reliable biomarkers that make therapy indication and success measurable. For this purpose, phenotypic characteristics must be chosen that are significantly associated with morbidity, such as eosinophils in sputum or FeNO in exhaled air. Even though several biologics are already available for eosinophilic asthma, many asthmatics still are non-responders. It might be possible that in these patients the individually relevant pathway is not addressed. Since asthma in its phenotypic and endotypic heterogeneity is to be understood as a syndrome, individualized therapeutic approaches can only be effective if the selected drug targets the dysregulated pathway of the individual patient. For the “T_H_2-low” endotypes there are no approved monoclonal antibodies so far. Only the compound tezepelumab has reached phase III – there still is a lot of room for improvement. 

## Acknowledgment 

We thank Elisabeth Pakic for preparing the figure. 

## Funding 

None. 

## Conflict of interest 

The authors declare no conflicts of interest. 

**Table 1. Table1:** Overview of asthma endotypes.

	**T** **_H_** **2-high**	**T** **_H_** **2-low**
Prominent inflammatory type in sputum	Eosinophil	Neutrophil Mixed-granulocytic Pauci-granulocytic
Relevant pathways	IL-4, IL-5, IL-13	IL-17 (?)
Approved biologics	Omalizumab (IgE) Dupilumab (IL-4, IL-13) Benralizumab (IL-5) Mepolizumab (IL-5) Reslizumab (IL-5)	–
Selection of investigational biologics (pathway)	Tezepelumab (TSLP) CSJ117 (TSLP)	
	Astegolimab (IL-33)	CJM112 (IL-17A)
	GSK3772847 (IL-33)	

**Table 2. Table2:** Profiles of approved asthma biologics.

Substance (year of approval)	Target	Application	Biomarker	Effect	
				Exacerbations	Cortisone use
Omalizumab (2005)	IgE	s.c.	IgE	↓↓	↓
Mepolizumab (2015)	IL-5	s.c.	Blood eosinophils	↓↓	↓↓
Reslizumab (2016)	IL-5	i.v.	Blood eosinophils	↓↓	?
Benralizumab (2018)	IL-5Rα	s.c.	Blood eosinophils	↓↓	↓↓
Dupilumab (2017)	IL-4Rα	s.c.	FeNO	↓↓	↓↓
